# Optimising burns referrals in response to COVID-19

**DOI:** 10.1016/j.jpra.2021.01.004

**Published:** 2021-01-29

**Authors:** Jack Jones, Jonathan J Cubitt

**Affiliations:** a Swansea University College of Medicine: Swansea University Medical School, Swansea, United Kingdom; b Welsh Centre for Burns and Plastics Surgery, Morriston Hospital, Heol Maes Eglwys, Morriston, Swansea, Wales, United Kingdom

*Dear Editor*,

The COVID-19 pandemic has driven both scientific and technological innovation and is thus forcing healthcare to adapt in order to provide an efficient and safe service during these times.^1^ For the burns specialty this has come in the form of tele-referral services. Although this particular technology was introduced prior to 2020, COVID-19 has driven the need for its use and indeed has accelerated its adoption.

The electronic-web based system enables clinicians to securely send images of patient injuries via a smartphone, meaning patients can now be triaged digitally prior to any face-to-face management. A study comparing the diagnostic accuracy of a telemedicine tool against a traditional face-to-face consultation reveals an extremely high sensitivity and specificity (99.4% and 100% respectively) of the digital service.^2^ Furthermore, findings of a report conducted into the impact of the technology at a UK Burns Centre detailed promising support for the technology with clinicians discussing the ease and accuracy of this new digital system.^3^ However, whilst anecdotal evidence has demonstrated great support for the tool, little quantitative evidence of the tele-referrals impact has been presented. Therefore, this report aims to look at both the quantitative and qualitative impact of a tele-referral service on a UK Burns Centre in the South West Network during the COVID-19 pandemic.

The retrospective study was conducted over a 2-month period from the introduction of the tele-referral service in July 2020. Patients were referred from 28 hospitals across the South West Burns Network. Included in the digital referral are basic patient identity details, the mechanism of burn injury, the total body surface area (TBSA) burn percentage and images of the injury.

During the 2-month period, 347 referrals were made: 152 (44%) were managed remotely and 195 (56%) were brought into hospital either for admission or for an outpatient appointment (Figure 1). Remote management of the 44% consisted of either; advice and treatment at the referring hospital, being seen by the Burns Centre outreach team, deemed inappropriate, no available bed, patient declined treatment or no burn.

**Figure 1:**
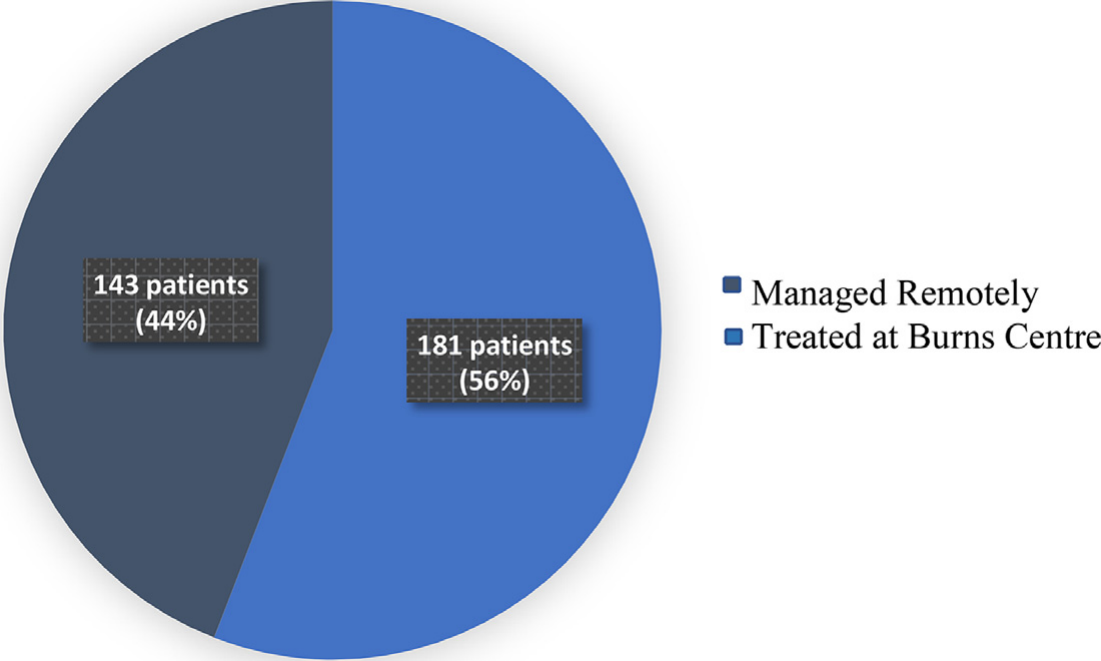
Chart demonstrating the number of patients that were managed remotely (44%) and the number that were treated at the Burns Centre (56%).

Before the introduction of the tele-referral service, patients would have been referred by telephone and would have all been seen in a face-to-face outpatient appointment at the Burns Centre. However, research has shown that TBSA burn percentage accuracy is routinely mis-estimated regardless of provider level, and as a result 77% of Burns Centre referrals are inappropriate. Therefore, a tele-medicine service that is both accurate and efficient is needed.^4^

Burns outpatient appointments are known to be approximately 50% more expensive than other outpatient appointments. Applying this to an estimated cost for an outpatient appointment at this hospital, it was calculated that over the first two months of the tele- referral service, a cost saving of approximately £15,808 was made for the Burns Centre (see fig including workings). This figure should also be viewed as a gross underestimation given it does not account for any consumables (e.g. dressings) that may have been required to treat the patient. From a qualitative perspective, fewer individual patients attending the Burns Centre means the expertise and resources can be focused on those patients that require it the most.

From a patient perspective, the subset who were managed remotely benefitted by being spared the journey for a face-to-face outpatient appointment at the Burns Centre. As one of the four regional adult UK Burn Centres, Morriston Hospital in the Swansea Bay Health Board serves a population of approximately 10 million.^5^ As a group, 77 miles were saved per patient. Whilst this may seem insignificant, some of these patients were referred from as far as Cornwall, which equates to a near 500-mile round trip.

In addition to the above hospital and patient benefits, the tele-referral system has been invaluable during the COVID-19 pandemic. The need to avoid unnecessary travel and minimise face-to-face contact has been paramount in limiting the spread of the SARS-COV-2 virus. The tele-referral service has provided a means of digitally triaging acute burns patients remotely, thus minimising footfall within the hospital.

The age of digital medicine is upon us, and now more than ever we are seeing its benefits. This report once again demonstrates the positive impact the tele-referral technology has had on patients and clinicians alike in providing a more efficient service for the referral of burn injuries. As has been suggested by previous studies, this technology could be used in other specialties, namely Trauma, Plastics and Dermatology, given the visual importance for those areas of medicine.^3^ Additionally, this technology can be used as an educational tool, given the number of injuries that will be photographically recorded. Finally, with the public health guidance surrounding the COVID-19 pandemic, there is an increasing need for patients to be managed remotely where possible. For burns, this tele-referral service has demonstrated it is an effective tool for doing so.

## Declaration of Competing Interest

None.
